# Evaluating the Feasibility of Implementing School Staff Well‐Being Programs

**DOI:** 10.1111/josh.70286

**Published:** 2026-11-01

**Authors:** Jimmy Duhamahoro, Angela M. Tuttle Prince, Spyridoula Vazou, Sally S. DuBois, Gregory J. Welk

**Affiliations:** ^1^ Department of Kinesiology and Health Iowa State University of Science and Technology Ames Iowa USA; ^2^ School of Education Iowa State University of Science and Technology Ames Iowa USA; ^3^ Department of Kinesiology Michigan State University East Lansing Michigan USA; ^4^ Iowa State University Extension and Outreach, Iowa State University of Science and Technology Ames Iowa USA

**Keywords:** feasibility, mixed methods, school staff, staff well‐being

## Abstract

**Background:**

Staff well‐being is a critical component of school wellness. This study leveraged the statewide School Wellness Integration Targeting Child Health (SWITCH) initiative to evaluate the feasibility of implementing supplemental staff well‐being programs.

**Method:**

Elementary schools that participated in two consecutive years of SWITCH (2022/2023 and 2023/2024) were invited to adopt staff‐focused initiatives through three structured programs: Alliance for a Healthier Generation, Make It OK, and 5‐2‐1‐0 Healthy Choices Count! Feasibility was assessed using survey response rates, program ratings, and semi‐structured interviews for demand, acceptability, and integration.

**Results:**

Sixteen schools (88%) expressed interest in Year 1 and eight (38%) in Year 2; six participated across both years. Seventy‐one percent of leaders rated programming quality as high and noted benefits such as increased staff support and attention to well‐being. Only 48% perceived improvements as sustainable, citing the need for ongoing resources and structural support to maintain long‐term implementation.

**Implications for School Health Policy, Practice, and Equity:**

Building school capacity, providing continual support, and leveraging existing wellness infrastructure can strengthen adoption of staff well‐being programming.

**Conclusion:**

Integrating staff well‐being initiatives into established school health frameworks is feasible and offers a foundation for broader implementation.

## 1 Introduction

High levels of work‐related stress are detrimental to employee well‐being and organizational functioning. School teachers face unique challenges, including persistent stress, anxiety symptoms, and depression [1]. Inadequate resources, high workloads, and student behavior challenges are among the most commonly reported sources of teachers' stress [2, 3]. The implications of these challenges for job satisfaction are well documented, particularly in their contribution to the national teacher retention crisis. More than 43% of K–12 teachers report intentions to leave the profession and a decreased enthusiasm for their work [3, 4]. High turnover rates and constant shifts in responsibilities further prevent teachers from achieving a well‐rounded work‐life balance. A statewide survey of Iowa superintendents found that 81.18% of open teaching positions in the state of Iowa for the 2023–2024 school year were filled by not fully qualified staff, resulting in increased workloads for existing teachers and a shortage of qualified instructors for desired courses [5]. Attending to teachers' well‐being has far‐reaching consequences—not only for their personal and professional functioning but also for instructional quality and the broader learning environment [6]. Collectively, these retention and staffing challenges have heightened awareness of the need to evaluate and improve well‐being in schools.

Despite a clear need for employee well‐being programs in schools, few systematic efforts have focused on developing programming specifically for school staff. Standard worksite well‐being interventions, such as health education, stress‐management training, and subsidized gym memberships, have shown positive effects on well‐being in isolated projects [3, 7]. Yet sustaining and scaling these interventions remains challenging due to the multidimensional nature of well‐being and the unique implementation needs of school settings [8, 9]. The limited research addressing these contextual factors underscores the importance of examining school environments alongside the design and testing of interventions. Such dual attention helps ensure that effectiveness is interpreted in light of critical implementation‐readiness factors, including capacity and motivation at both the individual and organizational levels [10, 11, 12]. However, implementation outcomes are often considered only after effectiveness trials [13], and new evidence frequently encounters barriers to adoption and programming when translated into practice.

Key factors that influence how well innovations are translated into practice include financial barriers, time constraints, the degree of adaptation required from original program models, and broader contextual implementation factors [7, 14]. In particular, the complexity of packaging and delivering effective strategies can overwhelm schools' capacity to implement health and well‐being initiatives, especially when institutional capacity for knowledge translation is limited [15]. Before testing and implementing public health initiatives at scale, it is therefore critical to assess implementation outcomes such as feasibility and adoption. In this context, the present study evaluated the feasibility of implementing school staff well‐being initiatives within an existing statewide school wellness program for youth—SWITCH (School Wellness Integration Targeting Child Health) [16, 17].

## 2 Methods

### 2.1 Participants

The SWITCH program recruits schools statewide before each academic year. School employees from elementary schools that participated in SWITCH for two consecutive years (2022/2023 and 2023/2024) were recruited for this evaluation. Schools were categorized into three levels based on programming needs and capacity. Level 1 schools were new to SWITCH and had 1–2 core team members (SWITCH unit leaders at each school, typically teachers, administrators, or staff). Level 2 schools had some prior SWITCH experience and a core team of 2–3 members. Level 3 schools were experienced sites with a stable team of three leaders, positioned to implement the full SWITCH program. All schools were incentivized ($200 per school) to participate in the supplemental staff well‐being program. Interested schools completed a staff well‐being evaluation survey in Year 1 and Year 2, and tested the implementation of staff well‐being enhancement initiatives in Year 2.

### 2.2 Instrumentation

Changes in school staff well‐being from Year 1 to Year 2 were assessed using an integrated Qualtrics survey that captured staff perceptions of their functioning, feelings, and performance over the previous 2 weeks [6]. The instrument included four domains—physical well‐being, personal well‐being, professional well‐being, and school climate—each with distinct response formats. Physical well‐being consisted of eight Yes/No items assessing physical activity, diet, sleep, and stress. Personal well‐being was measured using the 14‐item Warwick–Edinburgh Mental Well‐Being Scale (WEMWBS), a validated self‐report measure with strong psychometric properties (goodness‐of‐fit index = 0.93; Cronbach's *α* = 0.91; ICC = 0.83, *p* < 0.01) [18]. WEMWBS items capture both hedonic (feeling good) and eudaimonic (functioning well) aspects of well‐being. Professional well‐being was assessed through 14 items capturing staff feelings and thoughts at work, whereas school climate was measured using nine items reflecting perceptions of school culture and climate. Items for both domains were drawn from the Panorama staff and teacher survey [19]. The full integrated survey is provided in the supplementary materials (Table S1–S5).

To assess factors influencing implementation of the well‐being initiatives, tailored evaluation surveys were administered to school core teams at the end of Year 2 to capture perceived impact and satisfaction with implementation. Additionally, a script of open‐ended prompts was iteratively developed by all authors to guide semi‐structured interviews conducted at the conclusion of programming. Insights from quantitative and qualitative data were used to assess feasibility across key domains, including demand, acceptability, and integration within existing SWITCH structures, and early signals of sustainability.

### 2.3 Procedures

SWITCH is an evidence‐based implementation process used statewide to help schools create healthier environments through programs targeting youth health outcomes [16, 17, 20]. In this implementation cycle, schools were guided using the same process, but had the opportunity to engage in an additional layer of staff‐focused programming. The basic SWITCH implementation process is described first, followed by details on the release of the staff well‐being program. Schools that opt into SWITCH in the summer/fall were invited to attend an in‐person, day‐long training session led by Iowa State University Extension and Outreach/4‐H (ISU EO/4‐H) staff and the Iowa State University research team. The collaboration, based on principles from the interactive system framework [21], provides complementary lines of support to prepare teams to implement SWITCH during the upcoming academic year. Ongoing support and technical assistance for the promotion, launch and implementation of SWITCH is provided by ISU EO/4‐H staff throughout the entire school year. The implementation team provides support for promotion, implementation and evaluation of SWITCH using an array of communication channels (webinars, print resources, online posts, social media and email). This implementation process is continually tested and refined to build schools' capacity to implement their own programming [17, 22, 23].

These established SWITCH procedures provided the platform for implementing and evaluating supplemental staff well‐being programming. In Year 1, leaders from the Healthiest State Initiative (HSI) were invited to engage in the state‐wide Make It OK initiative, a mental health stigma‐reduction initiative [24]. The programming was shared as a supplemental support option for schools interested in staff‐focused well‐being activities. Team leaders from interested schools were encouraged to administer the staff well‐being survey as a baseline needs‐assessment tool.

In Year 2, schools that expressed continued interest were guided by HSI personnel to implement one of three community‐based, adult‐focused programs based on their needs: Make It OK, the Alliance for a Healthier Generation, or 5‐2‐1‐0 Healthy Choices Count! [24, 25, 26]. Each program provided modular resources that allowed schools to select strategies aligned with their local needs. Make It OK schools received a toolkit consisting of a series of educational videos to integrate mental health awareness into the workplace; Alliance for a Healthier Generation schools participated in monthly webinars focused on staff well‐being; and 5‐2‐1‐0 schools completed a needs‐assessment process to identify policy, systems, and environmental supports for staff well‐being, followed by tailored implementation guidance from HSI. Consistent with foundational SWITCH approaches anchored in self‐determination theory [27], schools had autonomy to choose what worked best to address their local needs and had support to enhance competence in delivering the programming.

### 2.4 Data Analysis

Data were analyzed using qualitative and quantitative methods at the school level, with findings integrated through a multimethod narrative synthesis.

#### 2.4.1 Quantitative Analysis

Data from the staff well‐being and program evaluation surveys were exported from Qualtrics in numerical format. Evaluation survey responses were summarized using frequency counts. Staff well‐being data required additional processing to place all items on a comparable 1–5 scale and to compute a composite score. All items were transformed so higher values indicated more positive well‐being. Physical well‐being items were reverse coded (“Yes” = better well‐being), and negatively worded professional well‐being items (3, 4, 7, 9, 10) were reverse coded using Newscore=6−oldscore. The eight binary physical well‐being items (1, 2) were linearly rescaled to (1–5) Physical wellbeing=1+4X(mean−1). After conversion, domain averages were combined to create an equally weighted composite well‐being score.

Because staff responses were nested within schools, linear mixed effects models were used to compare Year 1 and Year 2 well‐being. The composite score was the outcome, with survey year, sex, staff role, and years of experience as fixed effects and school as a random intercept. Given that different staff could respond each year, analyses were treated as repeated cross‐sectional comparisons. Secondary mixed effects models examined each domain separately using the same model structure.

#### 2.4.2 Qualitative Analysis

Semi‐structured interviews were administered, recorded, and transcribed anonymously by three authors. The second author completed all transcriptions, and the lead author independently coded and developed preliminary themes. To ensure credibility and trustworthiness, two additional authors cross‐checked the coding against the original transcripts, and the team met iteratively to resolve discrepancies and reach consensus on the final thematic structure. A coding manual outlining the final code structure is provided in the supplementary materials (Table S6).

## 3 Results

### 3.1 Implementation and Staff Well‐Being Programming

Sixteen of the eighteen SWITCH schools participating in youth programming in Year 1 (88%) expressed interest in adding the staff well‐being component, and all 16 administered the staff well‐being survey, yielding a 60% response rate from staff in their school. In Year 2, five returning schools shifted their priorities to youth programming only, and five discontinued SWITCH entirely, leaving six schools that continued staff programming by re‐administering the survey and piloting staff well‐being initiatives. Two schools new to SWITCH opted into staff programming for the first time, bringing the total to eight participating schools in Year 2 (38% of 21 SWITCH schools), with an 87% staff survey response rate. Across these eight schools, five implemented 5‐2‐1‐0 Healthy Choices Count!, two adopted Make It OK, and one used the Alliance for a Healthier Generation.

Implementation structures varied across initiatives and schools, reflecting efforts to align programming with local needs and capacity. The five 5‐2‐1‐0 schools (staff *n* = 151) emphasized physical well‐being through recurring nutrition and activity‐based tasks. Dosage included weekly and monthly resource sharing, regular email reminders, and use of staff lounges and bulletin boards for ongoing exposure. Schools reported weekly fruit distribution in partnership with local grocery stores, staff‐created fruit buffets, and interactive activities such as blender‐bike smoothie‐making sessions and well‐being bingo that incorporated physical activity, mindfulness, and social connection. The two Make It OK schools (staff *n* = 43) delivered a one‐time four‐module mental health awareness series supplemented by reflective discussions, case‐study evaluations, and contextualized action planning. These activities targeted personal, professional, and school‐climate dimensions of well‐being. The single Alliance for a Healthier Generation school (staff *n* = 18) implemented recurring stress‐management and activities spanning all four well‐being domains. Dosage included weekly relaxation and yoga sessions, structured opportunities for staff interaction, motivational programming, and after‐school or lunchtime physical activity offerings.

The summary of programmatic ratings from schools, collected at the end of Year 2, is shown in Figure 1. Satisfaction with the programming—both in terms of short‐ and long‐term impact—was generally moderate to high. Notably, 71% of core team leaders rated the quality of the programming process as high, and 71% also rated the perceived effectiveness of their implementation efforts as high. However, concerns emerged regarding sustainability: only a minority felt confident that the observed improvements in school culture would persist, with 43% rating sustainability as moderate and 14% as low.

**FIGURE 1:**
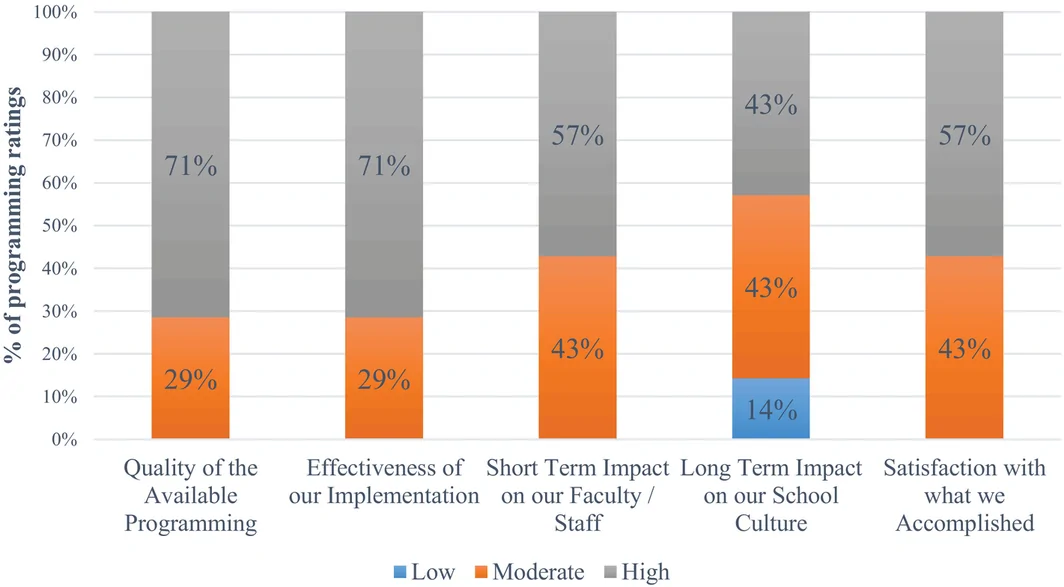
Staff well‐being program ratings from school core teams.

### 3.2 Factors Influencing the Implementation of Staff Well‐Being Programs

Interviews with core team leaders provided insight into the key facilitators and barriers influencing the adoption and implementation of the well‐being initiatives. Four main themes emerged from the analysis: motivation, dissemination, perceived benefits, and program structure (see Table 1). Core team leaders described several factors that motivated them to take on additional programming, including selecting initiatives that fit within their limited schedules and the desire to try a different option—particularly among those who had already experienced Make It OK resources in Year 1. Many leaders also emphasized self‐reflection and perceived benefits, such as improved productivity, as primary drivers of engagement in staff well‐being programming.

**TABLE 1:** Factors influencing staff wellbeing programming, based on interviews with school core team leaders.

Theme	Description
Motivation	*SN06:* “We are so focused on students we forget about ourselves, and we wanted our staff to know we were thinking of them and caring about them so that they could do their job to their best ability.” *SN15:* “It is important for us that we support the message that it's okay to not always be okay, to have an off day, to get rid of the stigma surrounding mental illness and finding a new way to approach it.” *SN09:* “I wanted to do the healthier generation one, but with scheduling of it and the hour meetings we can't make that work, we've had good feedback in the past. with that and they're open. We did the Make It OK, so it works well in the K‐12 building because a lot of the things apply to the kindergarten teacher that also apply to the college‐level math teacher so that's a good correlating factor there.” *SN13:* “The reason why I decided to do that one was that I was hoping for something different.”
Dissemination	*SN06:* “Every Friday to PD time to talk to them about it, did staff bingo, it was well received by most. Also sent emails about weekly health tips and how to get that in during the week, gave incentives with this.” *SN10:* “I brought it up to administration to have our well‐being policy pushed out to staff at the beginning of the year.”
Perceived benefits	*SN09:* “People are not feeling like it's okay for them to say they can't do or handle something, because it's their job, but some days they just want to go home and cry. This kind of helped us forge some new relationships with our coworkers because so many people had similar ideas and so learning that there are other people to talk to or even asking the administrators if you need a break from it or asking for support.” *SN06:* “Our staff members are competitive, so we wanted to amp it up, we looked at what worked and what didn't. We wanted to re‐emphasize what they were working toward, water consumption, step count, play a game with a family member, physical activity goals, mindfulness, and vegetable intake.” *SN10:* “Doing it was super easy for me because it does not take too much work, but it also makes a bigger impact than the work you put in.”
Program structure	*SN09:* “A lot of them when we got done and asked what's next, how to solve some of those problems to make it better, so a lot of people are open to working on that.” *SN10:* “In the weekly messages just giving a few examples of how other schools have been implementing similar things that we are trying to do would be good just for ideas for staff wellness.” *SN13:* “No, the staff wellness department had plenty of information. It is a matter of taking the information we got and figuring out what will work for this staff.” *SN15:* “More ideas of how to support the staff. We have coming up teacher appreciation, where one thing is parents will serve lunch, do recess duty, teachers can switch classrooms. The ambassadors suggested having volunteers to help with recess.”

*Note:* SN codes were used to reference anonymized transcript excerpts from interviews with school core team leaders.

Leaders outlined the dissemination channels they used to communicate program components to staff, noting that sharing information broadly and regularly updating colleagues were essential to maintaining engagement. Both structured approaches (e.g., routine email communication) and informal interactions (e.g., conversations during physical activity sessions or in staff lounges) were used. Consistent with SWITCH practices, leveraging champions and ambassadors played a central role in relaying implementation details. Leaders also noted anticipated benefits, such as increased staff cohesion and the ease of integrating activities, as factors that supported their continued involvement. At the same time, they expressed a desire for more structured and sustained delivery, along with clearer guidance on how to use available resources over time.

### 3.3 Staff Wellbeing Initiative Impacts

The impacts of programming were evaluated using the six schools that participated in both Year 1 and Year 2: three implementing 5‐2‐1‐0 Healthy Choices Count!, two implementing Make It OK, and one using the Alliance for a Healthier Generation. These schools represented six counties and were predominantly located in rural areas or rural pockets within larger districts. Two were classified as Level 2 and four as Level 3 within SWITCH programming. Over the 2 years, 337 staff participated (79% female, 75% teachers, 40% with fewer than 5 years of experience), with response rates of 76% in Year 1 (*n* = 165) and 81% in Year 2 (*n* = 172) (see Table 2).

**TABLE 2:** School staff characteristics from six schools that participated in staff well‐being implementation.

	Year 1		Year 2	
Characteristics	*n*	Percentage	*N*	Percentage
Sex				
Female	133	79.4%	142	82.6%
Male	32	18.2%	30	17.4%
Role				
Teachers	123	74.5%	114	66.3%
Faculty/staff	28	17.0%	42	24.4%
Administrator	8	4.8%	6	3.5%
Other roles	6	3.6%	10	5.8%
Experience at the current school				
< 5 years	66	40.0%	82	47.7%
> 20 years	34	20.6%	34	19.8%
11–15 years	24	14.5%	20	11.6%
6–10 years	23	13.9%	27	15.7%
15–20 years	18	10.9%	9	5.2%
Well‐being initiative implemented				
5‐2‐1‐0 (3 schools)			111	64.5%
Make It OK (2 schools)			43	25.0%
Alliance HG (1 school)			18	10.5%
Schools				
SN03	64	38.8%	42	24.4%
SN06	27	16.4%	34	19.8%
SN09	26	15.8%	28	16.3%
SN10	17	10.3%	35	20.3%
SN13	20	12.1%	18	10.5%
SN15	11	6.7%	15	8.7%

There was no statistically significant difference in overall well‐being between Year 1 and Year 2 (*β* = 0.08, 95% CI [−0.02, 0.19], *p* > 0.11). Among the four well‐being domains, only physical well‐being showed a significant school‐level increase (*β* = 0.24, 95% CI [0.06, 0.42], *p* < 0.007). No significant changes were observed in personal well‐being (*β* = 0.03, 95% CI [−0.09, 0.17], *p* > 0.59), professional well‐being (*β* = 0.07, 95% CI [−0.04, 0.20], *p* > 0.23), or school climate (*β* = −0.02, 95% CI [−0.14, 0.09], *p* > 0.11).

Exploratory analyses examining differences in overall well‐being by sex, staff role, and years of experience revealed no significant effects, with one exception: staff with more than 20 years at their school demonstrated a significant increase in overall well‐being (*β* = 0.19, *p* < 0.01). These findings did not change when an unrestricted sensitivity analysis of all 16 schools in Year 1 and eight in Year 2 was utilized instead.

## 4 Discussion

This evaluation demonstrated clear feasibility for introducing staff well‐being programming within the existing SWITCH infrastructure. Schools showed strong interest (88% engagement), and school teams followed guidelines to implement one of the three staff well‐being initiatives. Acceptability was reflected in consistently high staff survey response rates (an average of 60% across schools) and leaders' positive perceptions of the programming. A strength of the design is that schools opted into the supplemental faculty‐staff well‐being programming while also delivering the youth‐based programming for their students. The naturalistic implementation patterns demonstrate the overall feasibility of the programming as schools adapted activities to local needs and integrated them into existing communication channels and routines. The subsequent sections summarize the outcomes and explore the contextual factors necessary for long‐term adoption.

### 4.1 Participation and Engagement in Staff Well‐Being Programming

Schools that participated in the staff well‐being programming in Year 2 (38%) demonstrated strong engagement with the programming. Survey participation remained strong (87% response rate), and staff reported active involvement in planned activities. Schools valued the needs‐assessment process and were motivated to use available resources to reflect on local priorities and improve multiple domains of staff well‐being. Participation was higher among rural schools, which suggests they may have perceived greater value or need for such programming than urban schools. However, it is not clear whether enrollment was driven by incentives or intrinsic interest.

Schools new to the SWITCH program (categorized as Level 1) did not opt into the supplemental programming, a pattern which may be due to lower organizational capacity or readiness. Schools at Levels 2 and 3 appeared structurally equipped to integrate additional programming, whereas those new to the program may have been concerned about taking on too much. These observations align with previous SWITCH evaluations showing that implementation success is closely tied to organizational capacity and readiness, with higher‐capacity schools consistently reporting greater improvements in child and youth outcomes [22].

Sustaining school wellness programming is inherently challenging due to financial constraints, regulatory demands, and competing priorities operating across multiple levels—challenges common to most community‐based initiatives [28, 29, 30, 31]. Such factors help explain the difficulty of maintaining participation in real‐world settings, reflected here by only six schools continuing into both Year 1 and Year 2. Expanding the evaluation of staff programming within SWITCH would allow for more systematic assessment of factors influencing interest, enrollment, and implementation among early adopters, late adopters, and nonadopters, including Level 1 schools and those that discontinued participation.

### 4.2 Factors Influencing the Implementation of Staff Well‐Being Programs

Findings indicate that schools successfully implemented staff well‐being initiatives alongside the core SWITCH program. This aligns with prior research demonstrating that school staff can effectively implement supplemental social–emotional learning initiatives [32]. Importantly, the addition of supplemental programming does not detract from implementing best practices to address stress and mental health challenges; rather, it reinforces the need for such efforts [14]. Implementation success was reflected in high ratings of satisfaction, perceived impact, and overall programming quality. Motivation for participation was often rooted in a recognition of unmet staff needs, as illustrated by one core team member: “We are so focused on students we forget about ourselves, and we wanted our staff to know we were thinking of them…”.

Ease of implementation and perceived benefits were also notable, with 57% of respondents providing high ratings on these dimensions. In contrast, ratings for long‐term sustainability were more mixed, with many respondents indicating only low or moderate confidence in sustaining the outcomes. Team leaders' feedback emphasized a need for continued support and resources, with schools expressing interest in future programming (e.g., “More ideas of how to support the staff” and “A lot of them… asked what's next…”). The “Program structure” theme underscored the importance of sustained resources and structured support to maintain momentum. These perspectives are consistent with existing literature showing that improvements in school climate and organizational culture develop gradually and require long‐term investment. For example, Daily et al. documented incremental academic improvements associated with sustained climate initiatives over 3 years [33], and Springer et al. reported long‐term organizational shifts in employee satisfaction over 8 years [34]. Together, these findings reinforce that meaningful change in school environments depends on ongoing, sustained effort.

Schools demonstrated a clear understanding of the implementation process and identified actionable strategies, such as revising wellness policies, organizing weekly initiatives, and creating engagement tools, such as well‐being challenges. The collaborative model embedded within SWITCH likely contributed to the autonomy and competence observed in implementation [27]. Additionally, the use of theoretical frameworks, particularly self‐determination theory and interactive system framework, appears to have supported collaborative structures and fostered ownership of the process [21, 27]. Overall, the reported and perceived success of schools that implemented staff well‐being programs reflects the strength of the established SWITCH infrastructure. Future efforts should focus on strategies to engage less‐experienced schools and support the adoption of multilevel programming.

### 4.3 Staff Well‐Being Initiative Outcomes

There were no significant changes in overall staff well‐being or in the personal, professional, and school‐climate domains from Year 1 to Year 2. The only significant improvement occurred in the physical well‐being domain. Although the absence of a control group limits causal interpretation, many of the reported activities centered on physical activity and nutrition, either as primary strategies or as convenient vehicles for stress relief and social connection, which may help explain this pattern. Improvements in physical well‐being may also reflect synergy with the youth‐focused SWITCH programming, which traditionally emphasizes physical activity and healthy behaviors. These small and isolated effects align with prior literature, most notably a systematic review on the topic which reported modest but meaningful gains [35]. Variation in staff well‐being measures across studies limits between‐study comparability [6, 9, 19]. This evaluation combined validated scales into an integrated measure tailored to school staff, though further work is needed to assess the reliability and validity of this composite. The integrated survey reflects the evaluation's intent to prioritize feasibility over efficacy to support data‐informed practice in schools. Accordingly, these findings are best interpreted as evidence of practical utility and directional improvement in real‐world settings.

### 4.4 Implications for School Health Policy, Practice, and Equity

This feasibility study offers important insights into how staff well‐being programming can be integrated within existing school health and wellness frameworks. The findings suggest that schools and the broader system‐level entities that oversee organizational health programming can establish and reinforce staff well‐being initiatives by leveraging existing wellness structures. Schools interested in taking parallel steps with programming are encouraged to conduct an internal needs assessment as well as a formative evaluation of capacity prior to launching staff wellness programming. Capturing perspectives of staff in this regard is critical in fostering school‐level motivation and staff engagement. Implementers of staff well‐being initiatives are therefore encouraged to use formative, process, and outcome evaluations and to triangulate insights to address local needs.

### 4.5 Limitations

This feasibility study employed a multiple‐methods approach to evaluate the implementation of staff well‐being programming within a real‐world school context. However, several limitations should be considered. The small sample size and predominance of rural schools limit the generalizability of the findings. Additionally, only experienced schools (Levels 2 and 3) participated, restricting the ability to assess how capacity influences adoption. It is also important to recognize that schools independently manage both implementation and evaluation activities. Although this autonomy can be beneficial, it may have contributed to lower response rates by increasing staff burden. Variations in autonomy across schools and the specific effects of individual staff well‐being initiatives were not examined in depth. Instead, the study relied on a group‐level assessment of overall planning and execution. Further limitations include the absence of control groups and randomization, which are critical for establishing causal inferences. As such, findings should be interpreted as hypothesis‐generating. Overall, additional research is needed to rigorously evaluate effectiveness at scale and to better understand mechanisms supporting long‐term improvements in school staff well‐being.

## 5 Conclusions

Staff well‐being is a critical component of school wellness and health. This study demonstrates that staff well‐being programming can be integrated into existing school health frameworks, indicating potential for broader implementation.

## Funding

The Scanlan Center for School Mental Health funded this project.

## Ethics Statement

The study received approval from the Iowa State University Institutional Review Board (Protocol #14–651). The use of secondary, de‐identified school data qualified for a waiver of informed consent.

## Conflicts of Interest

The authors declare no conflicts of interest.

## Supporting information


**Table S1:** Physical well‐being.
**Table S2:** The Warwick‐Edinburgh Mental Well‐being Scale (WEMWBS).
**Table S3:** Professional well‐being (*overall feelings and thoughts*).
**Table S4:** Professional well‐being (*work and interactions with students and colleagues*).
**Table S5:** School climate.
**Table S6:** Interview coding manual.

## Data Availability

The data that support the findings of this study are available from the corresponding author upon reasonable request.
