# Supplementary material for: Evaluating the Feasibility of Implementing School Staff Well‐Being Programs
Source: J Sch Health. 2026 Nov 1;96(11):e70286. doi: 10.1111/josh.70286 (PMC13725206; doi:10.1111/josh.70286)
Supplement: Supplementary file 1 [file JOSH-96-0-s001.docx]

**Supplementary materials**

**S1. School Staff Well-being Survey**

**S1.1. Part 1 Physical Well-being**

*Please complete this simple checklist to rate your personal wellness behaviors. In responding, please rate your behaviors based on your lifestyle* ***over the past 2 weeks*** *and not on what you would like your behavior to be.*

**Table S1. Physical Well-being**

Do you get an average of 30 minutes of moderate intensity activity (5 days per week) or perform sports or vigorous activity at least 3 days per week? (Note: a combination of moderate and vigorous activity during the week)

- Yes
- No

Do you perform resistance exercise or perform activities like Yoga, Tai Chi, or other movement activities at least 2 times per week?

- Yes
- No

Do you usually eat at least five fruits and vegetables each day?

- Yes
- No

Do you make an effort to limit the amount of sugar, sweets, and processed foods in your diet?

- Yes
- No

Do you have a regular sleep habit and get seven to eight hours of sleep per night?

- Yes
- No

Do you generally go to sleep easily and sleep through the night?

- Yes
- No

Do you usually feel that you can manage all of the tasks required of you in a given day?

- Yes
- No

Do you have family and friends ready to help and support you if needed?

- Yes
- No

**S1.2. Part 2 Personal Well-being**

*The questions in this section will ask about your personal well-being. Please reflect on your overall feelings and thoughts related to your own mental health. Select the option that best describes your experience* ***over the past 2 weeks.***

The Warwick-Edinburgh Mental Well-being Scale (WEMWBS). Below are some statements about feelings and thoughts. Please tick the box that best describes **your experience of each over the last 2 weeks.**

**Table S2.** The Warwick-Edinburgh Mental Well-being Scale (WEMWBS)

| None of the time | | Rarely | Some of the time | Often | All of the time |
| --- | --- | --- | --- | --- | --- |
| I've been feeling optimistic about the future | o | o | o | o | o |
| I've been feeling useful | o | o | o | o | o |
| I've been feeling relaxed | o | o | o | o | o |
| I've been feeling interested in other people | o | o | o | o | o |
| I've had energy to spare | o | o | o | o | o |
| I've been dealing with problems well | o | o | o | o | o |
| I've been thinking clearly | o | o | o | o | o |
| I've been feeling good about myself | o | o | o | o | o |
| I've been feeling close to other people | o | o | o | o | o |
| I've been feeling confident | o | o | o | o | o |
| I've been able to make up my own mind about things | o | o | o | o | o |
| I've been feeling loved | o | o | o | o | o |
| I've been interested in new things | o | o | o | o | o |
| I've been feeling cheerful | o | o | o | o | o |

**S1.3. Part 3 Professional Well-being**

*Please reflect on your overall feelings and thoughts related to your work and interactions with students and colleagues. Select the option that best describes your experience* ***over the past 2 weeks.***

**Table S3. Professional Well-being (***overall feelings and thoughts***)**

During the past 2 weeks, how often did you feel	at work?

|  | Almost never | Once in a while | Sometimes | Frequently | Almost always |
| --- | --- | --- | --- | --- | --- |
| engaged | o | o | o | o | o |
| excited | o | o | o | o | o |
| exhausted | o | o | o | o | o |
| frustrated | o | o | o | o | o |
| happy | o | o | o | o | o |
| hopeful | o | o | o | o | o |
| overwhelmed | o | o | o | o | o |
| safe | o | o | o | o | o |
| stressed out | o | o | o | o | o |
| worried | o | o | o | o | o |

Please select the option that best describes your experience **over the last 2 weeks.**

**Table S4. Professional Well-being (***work and interactions with students and colleagues***)**

|  | Not at all | Slightly | Somewhat | Quite | Extremely |
| --- | --- | --- | --- | --- | --- |
| How effective do you feel at your job right now? | o | o | o | o | o |
| How much does your work matter to you? | o | o | o | o | o |
| How meaningful for you is the work that you do? | o | o | o | o | o |
| Overall, how satisfied are you with your job right now? | o | o | o | o | o |

**S1.4. Part 4 School Climate**

*Please provide your current perceptions of the school culture and climate by selecting responses for each of the questions below.*

**Table S5. School Climate**

| Not at all | | Slightly | Somewhat | Quite | Extremely |
| --- | --- | --- | --- | --- | --- |
| On most days, how enthusiastic are the students about being at school? | o | o | o | o | o |
| To what extent are staff trusted to work in the way they think is best? | o | o | o | o | o |
| How positive are the attitudes of your colleagues? | o | o | o | o | o |
| How supportive are students in their interactions with each other? | o | o | o | o | o |
| How respectful are the relationships between staff and students? | o | o | o | o | o |
| How optimistic are you that your school will improve in the future? | o | o | o | o | o |
| How often do you see students helping each other without being prompted | o | o | o | o | o |
| When new initiatives are presented at your school, how supportive are your colleagues? | o | o | o | o | o |
| Overall, how positive is the working environment at your school? | o | o | o | o | o |

**S2. Interview Coding Manual**

The lead author (JD), who has formal training in qualitative and mixed‑methods research through MPH coursework and doctoral training, conducted the initial coding and theme development. The second author (ATP), an assistant professor with expertise in education and behavioral research, transcribed the interviews and iteratively verified the coding. The third author (VS), an assistant professor with training in qualitative methods within kinesiology, also reviewed and verified the coding and thematic integration. The fourth author (SDS), an educator and university extension professional, coordinated interviews and contributed contextual insight. Coder training and reliability were addressed through iterative consensus meetings among the first three authors, during which codes were refined, discrepancies resolved, and thematic interpretations aligned.

**Table S6. Interview Coding Manual**

| **Interview questions (Staff well-being supplemental programming)** | **Raw data** | **Codes** | **Categories** | **Themes** |
| --- | --- | --- | --- | --- |
| **What were some of the reasons your team decided to take on this programming?** | **SN03:** - I think one was that we focused on students for so long that we also know that staff stress is high too and we wanted to provide support there. I think sometimes teachers are forgotten about and the things that we did were super easy. | - *The need to pause and reflect* - *Tapping into available resources and support* - *Easy implementation* | - Existing motivations - Perceived benefits - Need-based - Opportunity | **Motivation** |
|  | **SN04:** - We have a need. It’s been a very stressful year at xxxxxxxx. We have had a lot of incidents come up that have made teaching life difficult. We knew we had a need and we have also struggled over the years with culture in our building. I think some of that is kind of unavoidable like if you are a 6^th^ grade teacher, your prep and your lunch and your free time does not line up with a kindergarten teacher. So, we did the blender bike on a PE day so that people could be together and do it with some of the coworkers that you don’t get to see very often just to kind of boost that staff moral across the board because there are people in this building that I haven’t seen in weeks. I feel. Like when we started this, around mid-March when we got this going, we felt that food and wellness would be the best way to reach people and it would be an easier way for us to accomplish our goal of doing so. | - *Opportunity and needs to address stress* - *Consistent stressors* - *Secondary gains* |  |  |
|  | **SN06:** We are so focused on students we forget about ourselves, and we wanted our staff to know we were thinking of them and caring about them so that they could do their job to their best ability. | - *Increase awareness and personnel upkeep* - *Targeting productivity* |  |  |
|  | **SN09:** I wanted to do the healthier generation one, but with scheduling of it and the hour meetings we can’t make that work, we’ve had good feedback in the past with that and they’re open. We did the make it okay so it works well in the k-12 building because a lot of the things apply to the kindergarten teacher that also apply to the college-level math teacher so that’s a good correlating factor there | - *Initiative characteristics* - *Availability of options* - *Trying something new* |  |  |
|  | **SN10:** I felt like taking that one on just because of my previous district compared to this district was so much more ahead of staff wellness. At xxxxxx we were more aware of staff wellness, so when I came here none of the things that were pushed there were pushed here. I felt like maybe they weren’t even aware of staff wellness being a thing so that’s why I wanted to push it. I would just walk through the hallways and see several teachers or para educators carrying a pop or little things like that, that would trigger me to think that we need to act on what I am teaching in the classroom. I have always been a big advocate for brain breaks, which I know is not technically staff, but I wanted other staff to be aware that it is good for their physical and mental wellbeing. I didn’t have any education on the wellness policy here at xxxxxx whereas at my previous district, the wellness policy was pushed out every year to all staff and we had to read it and make sure that we understood it and we knew who was on the team and they would get together every quarter and modify it. | - *Peer and influence* - *Champions* - *Previous experience* |  |  |
|  | **SN13:** - The reason why I decided to do that one was that I was hoping for something different. We have done the 5120 and have done some stuff with them in the past. I went through the “make it okay” training last year with my staff. Since we have done both of those options, I wanted to do something different. One of the sessions was on staff workrooms and how we can make that better. There was also a lot of neat information about gratitude. One of the sessions that I did was about filling your cup. It was about burnout and how to prevent it among teachers and administrators. | - *Trying something new* - *Familiarity of programming* - *Need-based implementation* - *Existing infrastructure and experience* |  |  |
|  | **SN15:** It is important for us that we support the message that it’s ok to not always be ok, to have an off day, to get rid of the stigma surrounding mental illness and finding a new way to approach it. It also helps us with some of the things with the kids too, knowing there’s more to the story than what they walk in the door with | - *Stress management reflections* - *Resolutions* - *Potential side benefits* |  |  |
|  | **SN18:** - I like to try to be healthy, so I wanted to push that on other people so that's kind of why we took that route with that. So, with that, I implemented wellness Wednesdays. Every Wednesday, staff can wear comfortable clothing. I also revamped our soda machine in the staff lounge, so I put more healthy options in them. We put a Powerade option, a body Armor option, Diet Coke, Coke Zero, Sprite, Dr. Pepper, things like that. So some more healthy options without it just being strictly pop. I also reached out to different grocery stores in Ames, and they donated food to us. On the next early out which is May 1^st^, we are doing the blender bike so we will do that for our PD. During our other PD times, some staff would get together and do different wellness things like doing a prayer ring or walking around the school. | - *Leadership and opportunity* - *Social gatherings* - *Healthy living* - *Intentions and motivations* |  |  |
| **Can you describe a bit more how you shared the concepts with faculty and staff in your school?** | **SN03:** - We had the ambassador go shop at the local grocery store for fruits and yogurts for our parfait bar and then we sent an email out to staff saying go enjoy a little snack. We had a ton of stuff, and it was all gone so I know people went back for more all throughout the day. Our classroom teachers sent out stuff all SWITCH long about how to be mindful and how to help yourself. I even think that zoom that we did with the 5120 helped me really figure out what brings me happiness. Sometimes as an adult it is sometimes hard to think of what those things could be | - *Synergy between information in relay in both youth and staff programming* - *Asynchronous written communication* - *Synchronous and didactic* | - Structured and unstructured channels of delivery - Context-informed delivery (administration,   Familiarity of the program)   - Capitalizing on existing structure (SWITCH modules and ambassadors) | **Dissemination** |
|  | **SN04:** - We had eight weeks of Wednesday where we had fruit to create a fruit buffet in the teachers’ lounge just to kind of get the idea of bringing fresh fruits in and then every other week, so there were four weeks we brought four different recipes of infused waters and had those available in the teachers’ lounge. One day we had the blender bike, another day we used one of the quick bites recipes which was the popcorn trail mix and then for upcoming things we are having a charcutier board challenge to challenge teachers to put together healthy foods and healthy dips. | - *Regular schedule and structured gatherings* - *Team building activities* |  |  |
|  | **SN06:** Every Friday to PD time to talk to them about it, did staff bingo, it was well received by most. Also sent emails about weekly health tips and how to get that in during the week, gave incentives with this. | - *Regular weekly meetings* - *Asynchronous written communication* |  |  |
|  | **SN09:** During an in-service. We broke off into small groups and had good conversations there. | - *Direct face-to-face conversations* - *Group-based discussions* |  |  |
|  | **SN10:** I brought it up to administration to have our wellbeing policy pushed out to staff at the beginning of the year. I know everyone is busy, but I suggest that each staff member highlight a few things that they think are important to staff to recognize and to form some type of committee around wellness.  - Bringing attention to the wellness policy for our staff to recognize and be aware of. I felt like it was not prepared to be seen by everyone. The discussions of modifying it happened behind the scenes, so I feel like next year, after it is edited to push that out to staff more and have something more surround the wellness policy of staff being educated around it. I also talked to Mr. Howard (administrator) about having something in there with staff drinks and incorporating that into the wellness policy. **Follow Up:** What did you do with the 5120 then?  That was centered around the mental well-being of the staff because I wanted to incorporate what we were doing in the classroom but push it out for staff. One of the more readably available things was the staff quotes in the lounge. Every week I would update a positive message for the staff on the bulletin board. Then before I did that, I sent out an email just to keep an eye out for it so that they kind of knew what it was for and who was doing it. I also hung the posters in the lounge as well and there was one time I brought a little snack with a mental wellbeing quote on it. | - *Planning and defining the structure of delivery* - *Involvement of higher ups for maximum impact* - *Unstructured conversations about programming* - *Asynchronous motivational messages delivered* - *A one-to-many delivery* |  |  |
|  | **SN13:** - Answered in question about |  |  |  |
|  | **SN15:** We sent those out to staff, and quite a few of the teachers responded and answered the questions, and I passed those on a document to xxxxxx [support team personnel] and she said that worked, we answered the questions and watched the videos. A lot of teachers it was sent out to who attended the presentations last year, kind of as a recap and comment on the videos. | - *Asynchronous channels* - *Communication based on the familiarity of the program* |  |  |
|  | **SN18:** See above |  |  |  |
| **Reflecting on the implementation, what key lessons have been learned?** | **SN03:** - I think our staff is always appreciative like xxxx said of attention, a kindness in some way. We have expressed sharing the positives and share what you do just so they feel seen and heard. | - *Moments and space for reflection as result of programming* - *Relatedness and cohesion from implementation* | - Staff programming related benefits at individual and group level - Reflections on the barriers and facilitators in implementation process | **Perceived benefits** |
|  | **SN04:** - I think for a lot of people, the infused water surprised them at how easy of a switch that is to make your water taste a little bit better. There are a lot of pop drinkers in our building, me being one of them. I try to drink water but it's just water, so having that infused water with that little extra flavor really did help me drink more throughout the day. It’s an easy thing to do. I had no idea what basil tasted like, and we had basil watermelon water one day so that was interesting because it sparked conversation with the staff and got us to try something new as well. A lot of people also said that they wanted their water cold and that was a big thing so I thought maybe we could use some of our money towards getting an ice machine if that is what is holding teachers back from drinking water more often.  - I think that the blender bike was a hit and when we put spinach in it, people were resentful of that, but you really couldn’t taste it, but it was interesting to look at all the nutritional value that you get out of that. I also know a lot of the teachers really liked the try day Friday. I think it was just a good time to bond with their students and make some of those connections with them. | - *Relative ease in implementation and behavior change* - *Easy to follow instructions* - *Increased awareness of personal habits* - *Increased motivations to build healthy habits* - *Created agency to find practical solutions* - *Reflection on the effects of programming on school climate* |  |  |
|  | **SN06:** Our staff members are competitive, so we wanted to amp it up, we looked at what worked and what didn’t. We wanted to re emphasis what they were working towards, water consumption, step count, play a game with a family member, physical activity goals, mindfulness, and vegetable intake. | - *Trialed Implementation and strategies* - *Provided goal-oriented guidance* |  |  |
|  | **SN09:** With our professions we often worry so much about our kids, but what are we doing for ourselves? People are not feeling like it’s okay for them to say they can’t do or handle something, because it’s their job, but some days they just want to go home and cry. This kind of helped us forge some new relationships with our coworkers because so many people had similar ideas and so learning that there is other people to talk to or even asking the administrators if you need a break from it or asking for support. | - *Increased relatedness, cohesion, and camaraderie* - *Built and strengthened a sense of community and belonging* |  |  |
|  | **SN10:** - Doing it was super easy for me because it does not take too much work, but it also makes a bigger impact than the work you put in. Just realizing that it is needed and necessary even if it is just a small gesture. Also taking note that the staff does need moral and mental boosters, so producing more strategic ways to implement it in the future is necessary. | - *The implementation ease and high return from relatively minimal efforts* - *A necessary factor in staff productivity* - *Reflection on streamlining the implementation* |  |  |
|  | **SN13:** - “What is your why” talked about how we are supposed to focus on what our why is and use those on the rough days and we did that with the whole staff. In the staff lounge we created a “what is your why” wall. I put a heart in everyone's mailbox so that they could put what their “why” is. We posted all of them in the lounge on the Bulletin board because it is all anonymous. | - *Implementation of the program created space for individual and group reflections* - *Brought staff together* |  |  |
|  | **SN15:** The general census was that it was appreciated to have that available. Some of the staff felt we could do a better job of supporting the wellbeing of the staff. Being a smaller community, we do a pretty good job of that, but towards spring break and the end of the year we lose the connect and it all ends up being fast. Being all through the year would be good, it’s really hard to stay connected and supported when things are happening, to have outlets for that and availability for teachers to have someone to talk to or connect with in our community of staff, not overwhelmed with stuff would be good.  We did some classes people could sign up for, some things in the community that staff could sign up for, we did a secret Santa but a “thankful for you” project, an ice cream social, this year wasn’t as great as previous years but just to get together as a group is good. These were something they could sign up for outside of school, those classes in the community | - *Exposed the need for staff programming and key areas/timing for interventions* - *Acknowledging the importance of having a well-rounded workforce* - *Creating programming options for different staff well-being needs* - *Comparative reflections on implementation from different years.* |  |  |
|  | **SN18:** - They never want wellness Wednesday to go away. They really enjoyed the fresh fruits. If there's food in the teachers' lounge, they will eat it so if it's healthier than it's better. I did get a lot of feedback. I asked for suggestions for the soda machine and the feedback from that was nice too.  - Our instructional coach sent out a day that we could go bike on the trail in xxxx (place) as well.  **Follow Up:** Do you feel that the adult well-being option was helpful with the reduction of stress of what were your thoughts on that.  - The ability to wear sweatpants on Wednesday was the best thing to happen. Staff love it so much and they look forward to it each week. At first, we were just doing this early on Wednesday, but we realized that we needed this every Wednesday.  **Follow Up:** You all worked with 5120, right?  - Yes | - *Enjoyment and fun from programming activities* - *Feedback as key strategy in the implementation* - *Acknowledged the role of instructional support and guidance* - *Reflected on behavior change enabling factors from the implementation and programming* |  |  |
| **How can we help you be more successful?** | **SN03:** - You guys provided a lot of great resources that we were able to pull from and put into play. I will say the one thing I missed was the family newsletter because I could just copy and paste and throw it out to our parents who were more engaged in what we are doing. I felt like my communication with parents this year was shortened because I was giving a 1-2 sentence explanation of a resources that I had pulled out and then advertising our cooking school. I don’t know if our parents missed that email because I don’t know how many of them were actually reading it, but I felt like I had more outreach. | - *Positives on programming and resource options* - *The need to expand the staff community through continuous parent engagement* | - Guidance on resource utilization - Sustaining and building on working strategies - Programming sustainment - Program success evaluation strategies - Need-based programming | **Program structure** |
|  | **SN04:** - Just giving us different ideas. I liked it when we met and got together and bounced ideas around from others. I also liked the sharing Facebook page to get ideas and of course the money doesn’t hurt, like that is a definite help is to have that extra money because school budgets are kind of an issue.  - We are doing some planning for stuff next year and one of the things we are planning is to have a school wellness team | - *Highlighted the need for novelty in resources* - *Emphasizing the need-based implementation strategies through consensus building.* - *Resolutions to establish a wellness team* - *Sustaining monetary incentives* |  |  |
|  | **SN06:** More tips and facts on newsletters geared towards the staff/adults. The staff like trinkets, maybe get gift bags for the staff. | - *The need for a continuous flow of resources on staff wellbeing programming* |  |  |
|  | **SN09:** A lot of them when we got done asked what’s next, how to solve some of those problems to make it better, so a lot of people are open to working on that | - *Highlights on the needs for service and program continuity through structural guidance and support* |  |  |
|  | **SN10:** - In the weekly messages just giving a few examples of how other schools have been implementing similar things that we are trying to do would be good just for ideas for staff wellness. | - *Providing schools with evaluation insights for comparative programming and use of resources* |  |  |
|  | **SN13:** - No, the staff wellness department had plenty of information. It is a matter of taking the information we got and figuring out what will work for this staff. | - *Satisfied with the provided resources, support, and actual staff programming done* - *Hints on structural approaches to using programming resources* |  |  |
|  | **SN15:** More ideas of how to support the staff. We have coming up teacher appreciation, where one thing is parents will serve lunch, do recess duty, teachers can switch classrooms. The ambassadors suggested having volunteers to help with recess. | - *Voiced the need for more programming supports* - *Supporting schools in the creation of comprehensive programs involving staff and parents* - *The need for support for staff recess during recess* |  |  |
|  | **SN18:** - I think if we dived deeper into it too because it was another one of those things that we were trying to do when we were trying to pick up the pieces from being shaken up.  - Another thing would be to ask for more staff input on things. An idea from a staff member was learning about water intake and then maybe implementing a competition and then have a prize with it  - I think the accountability with the people involved is something we hope to improve on for next year | - *Ensuring a need-based assessment prior to programming* - *Highlighting the need for sustained motivation and self-sustaining implementation.* |  |  |
